# Application of Trehalose Mitigates Short-Styled Flowers in Solanaceous Crops

**DOI:** 10.1021/acs.jafc.2c08479

**Published:** 2023-04-03

**Authors:** Izumi C. Mori, Takakazu Matsuura, Masahiro Otao, Lia Ooi, Yasuyo Nishimura, Takashi Hirayama

**Affiliations:** † Institute of Plant Science and Resources, Okayama University, Kurashiki 710-0046, Japan; ‡ Research Department, Hayashibara Co., Ltd., 675-1 Fujisaki, Naka-ku, Okayama 702-8006, Japan; § Faculty of Agriculture and Marine Science, Kochi University, Nankoku 783-8502, Japan

**Keywords:** solanaceous crops, short-styled flower, trehalose, maltose, plant biostimulation, biostimulant

## Abstract

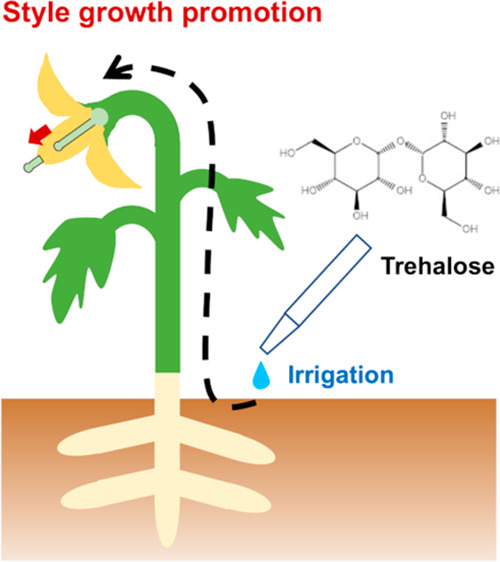

Trehalose is a disaccharide and is often foliar applied by farmers aiming at increasing stress resistance or crop production. However, the physiological effect of exogenously applied trehalose on crops remains obscure. Here, we explored the effect of foliar trehalose application on style length of solanaceous crops, *Solanum melongena* and *S. lycopersicum*. Trehalose application promotes pistil to stamen ratio by gaining style length. Another disaccharide consisting of two glucose molecules, maltose, showed the same effect on style length of *S. lycopersicum*, while monosaccharide glucose did not. Trehalose is found to affect style length through uptake via roots or interaction with rhizosphere but not through absorption by shoots in *S. lycopersicum*. Our study suggests that yield improvement of solanaceous crops by trehalose application under stressed conditions is brought about by suppression of the occurrence of short-styled flowers. This study suggests that trehalose holds potential to act as a plant biostimulant in preventing short-styled flowers in solanaceous crops.

## 1 Introduction

A short-styled flower is a flower that has a shorter pistil than stamens. It is an agronomical problem in solanaceous crops. As flowers of solanaceous crops are downward-facing, pollens could not reach to the stigma located inside the anther cones in a short-styled flower, and hence the pollination efficiency is lower. High temperature, insufficient light intensity, and nutrition deficiency are known to cause short-styled flowers in solanaceous crops.^1^ In cultivation practices for tomatoes (*Solanum lycopersicum*) and eggplants (*S. melongena*), farmers often employ chemical agent-mediated parthenocarpy to promote fruit set. Bell pepper (*Capsicum annuum*), however, sets fruits with abnormal shapes by parthenocarpy and requires successful pollination for fruit production. Therefore, prevention of short-styled flowers is critical for bell pepper cultivation and beneficial for other solanaceous crops, such as tomatoes and eggplants.

Trehalose is a disaccharide consisting of two glucose molecules joined by an α,α-(1 → 1) linkage. In the industry, trehalose is extensively utilized as food additives and cosmetic ingredients. In nature, it is naturally distributed across kingdoms of life.^2^ Trehalose accumulates under various stresses in many species as a compatible solute, while some species use it for energy metabolism. Its rich accumulation is the key for strong dormancy characteristic under extreme drought environments of some exceptionally drought-tolerant species, such as sleeping chironomids and resurrection plants.^2,3^ Nonetheless, the majority of plants contain only a very low concentration of trehalose. In plants, the role of trehalose-6-phosphate (T6P), the precursor of trehalose biosynthesis, has been established as a signal molecule for sucrose availability.^4^ However, the physiological roles of trehalose have not been well elucidated and remain debatable.

It was reported that exogenously applied trehalose showed some favorable effects in plants, strengthening resistance to pathogens, drought, and temperature stresses, while it shows detrimental effects on the vegetative growth (for review, see ref (5)). Farmers use trehalose aiming at mitigating the impact of stresses on crop production, suggesting its role as a biostimulant.^6^ Although its benefit has empirically been acknowledged by farmers, the physiological basis of the action of trehalose on crops has not been well understood. Precedential to this study, we observed an increment in fruit number of bell pepper by foliar application of trehalose.^7^ Here, we examined the effect of trehalose on crop yield and short-styled flower occurrence in Solanaceae crops to explore the physiological basis for beneficial effects of trehalose on solanaceous crops via field experiments (for eggplants) and controlled-environment experiments (for tomatoes).

## 2 Materials and Methods

### 2.1 Chemicals

High purity trehalose (99.2%) was provided by Hayashibara Co., Ltd. (Okayama, Japan). Maltose [21116-92 (≥98% purity)] and glucose [16806-25 (≥99% purity)] were guaranteed reagent products procured from Nacalai Tesque Inc. (Kyoto, Japan). Other chemicals were analytical grade from Nacalai Tesque Inc., Sigma-Aldrich (St. Louis, Missouri, United States), or Fujifilm Wako Pure Chemicals Corp. (Osaka, Japan).

### 2.2 Plant Growth and Sugar Treatments

#### 2.2.1 Eggplant Culture in a Greenhouse

Eggplants (*Solanum melongena* cvs. ‘Ryoma’, ‘Senryo-nigou’, ‘Kurobee’, ‘Kurowashi’, and ‘Nagaokanaga’) were cultivated in soil containing bark manure (3t 10 a^–1^), slow-release compound fertilizer (25 kg nitrogen 10 a^–1^, CDU555, Zen-noh) and dolomite lime (150 kg 10 a^–1^) at a 50 cm distance between seedlings, in a greenhouse on Monobe campus of Kochi University (N33.553, E133.678), in summer seasons. In the 2017 season, seedlings of ‘Ryoma’, ‘Kurowashi’, and ‘Nagaokanaga’ were transplanted in soil on April 19th after being in a nursery from February 27th in the greenhouse. In the following years, seedlings of ‘Ryoma’ were planted in soil on May 11th, 2018, and May 13th, 2019, after a 6-week nursery cultivation. For grafting, ‘Ryoma’ scions were grafted to the rootstock (cultivar ‘Daitarou’) on March 6th and planted in soil on May 2nd, 2019. Scions of ‘Senryo-nigou’ and ‘Kurobee’ were grafted to the rootstock (*S. melongena* cultivar ‘Daitarou’ or *S. torvum* cultivar ‘Torvum Vigor’) on April 10th and 11th, respectively, in 2018 and on March 1st, 2019, and planted in soil on May 11th, 2018, and May 2nd, 2019. Ridge width and planting distance were 1.5 and 0.5 m, respectively. Grafted seedlings were procured from Berg Earth Co., Ltd. (Uwajima, Japan). Seedlings without grafting were grown from seeds on Monobe campus of Kochi University.

#### 2.2.2 Trehalose Treatment of Eggplants

Whole shoots of eggplants were treated with trehalose solution [0, 0.05, or 0.1% (w/v)] prepared in water by foliar application once a week in the greenhouse from May 8th to July 24th in 2017, May 24th to August 9th in 2018, and May 8th to August 14th in 2019, respectively. Application of trehalose solution was done on both abaxial and adaxial sides of the leaves by spraying until the solution dripped down from the leaves (100 to 250 mL per plant).

#### 2.2.3 Tomato Culture in an Environment-Controlled Growth Chamber

Seeds of tomato (*S. lycopersicum* cultivar ‘Micro-Tom’) were imbibed with distilled water for 1 h in a Petri dish and successively sown in soil consisting of vermiculite (middle granule size, Nittai Co., Ltd., Osaka) and potting soil (Metro Mix 350J, Hyponex Japan Corp., Ltd., Osaka) at a 3:1 ratio in a plastic pot (0.23 L). Seedlings were thinned out to retain a healthy seedling per pot after 1 week of culture in an environment-controlled chamber (Biotron LPH-410SP, Nippon Medical & Chemical Instruments Co., Ltd., Osaka) at a day–night cycle of 16 h light (average photon flux density = 120 μmol m^–2^ s^–1^) at 25 °C and 8 h dark at 16 °C. Relative humidity was maintained at approximately 60% during the culture. Seedlings were successively cultured in the chamber for 8 weeks. Plants were watered every 2–3 days and fertilized with 1000-fold diluted Hyponex nutrient solution (6-10-5, Hyponex Japan Corp., Ltd.) once a week.

#### 2.2.4 Sugar Treatments of Tomato

Shoots of tomato plants were sprayed with aqueous solution containing trehalose (0, 0.1, and 0.5% (w/v)), glucose (0.5% (w/v)), or maltose (0.5% (w/v)) using a plastic sprayer, unless otherwise indicated. Approximately 5 mL of sugar solution was sprayed per plant once a week at a given concentration. The sugar treatments were started at the 3rd week after imbibition when the first true leaf expanded. The control treatment was carried out with distilled water.

Where indicated, 5 mL of sugar solution was applied to the soil with a glass pipet carefully avoiding direct contact of the solution to the shoots. Alternatively, a sugar solution was applied to the leaves or inflorescence using a paint brush while covering the surface of the pot with plastic film to avoid dropping of the sugar solution to the soil.

### 2.3 Measurements of Flower Morphology

#### 2.3.1 Short-Styled Flower Occurrence in Eggplants

The flowers of eggplants were visually classified into three classes, short-styled, medium-styled, and long-styled (Supplementary Figure S1). A medium-styled flower is a flower with the tip of the anther cone and the tip of the stigma located at the same height. A long-styled flower is a flower with a protruded stigma from the tip of the anther cone. A short-styled flower is a flower with the stigma located beneath the tip of the anther cone. The classification of flower morphology was done grossly once in 2 weeks in 2017 and 2018, and every 2–3 days in 2019 through the culturing seasons.

#### 2.3.2 Style, Ovary, and Stamen Lengths of Tomato

**Figure 1:**
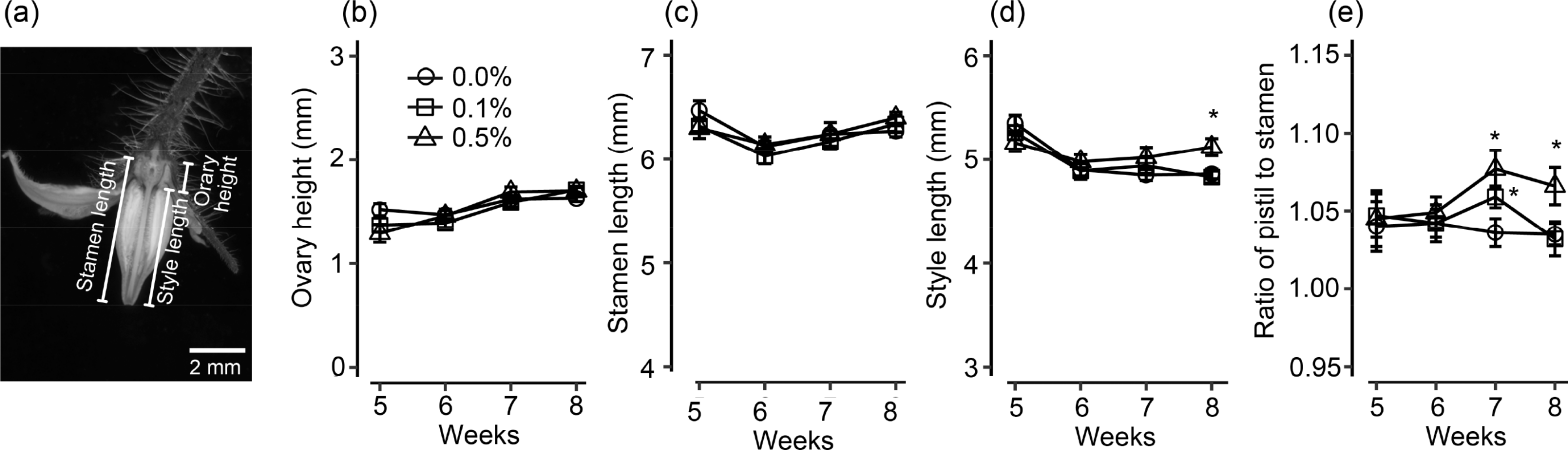
Effect of foliar application of trehalose on ovary height, stamen length, style length, and ratio of pistil length to stamen length of tomato (cultivar ‘Micro-Tom’). (a) Dissection of tomato flower for measurements. (b) Ovary height. (c) Stamen length. (d) Style length. (e) Ratio of pistil to stamen. Error bars represent standard deviation. Circle, square, and triangle indicate 0.0%, 0.1%, and 0.5% trehalose treatment, respectively. Asterisks indicate significance at α = 0.05 by Dunnett’s test compared to 0.0% trehalose. *n* = 9–11, 16–26, 14–30, and 20–33 flowers for 5th, 6th, 7th, and 8th week, respectively; derived from four independent experiments.

Flowers newly bloomed in the last 16 h were detached at 6 to 7 h after the onset of the light period every day. Lengths of style, ovary, and stamen were measured under a stereomicroscope (MVX10, Olympus Corp., Tokyo) after a manual dissection along the longitudinal axis of the flower with a scalpel (Figure 1a).

#### 2.3.3 Measurement of Pollen Germination Rate of Tomato

Immediately after the measurement of the lengths of style, ovary, and stamen, pollens were collected from the same flower on a glass slide by gently tapping the anthers with a pair of forceps. Collected pollens were suspended in 5–10 μL of 0.44 M sucrose solution with a micropipette and dropped on a film of 0.8% agar (2–3 mm thickness) containing 0.44 M sucrose in a Petri dish and successively incubated at 25 °C for 24 h. Germinated pollens were examined under a microscope after staining with 0.1% Safranin O. Pollen bearing a pollen tube of which length exceeded the diameter of the pollen was scored as a germinated pollen.

### 2.4 Quantification of Plant Hormones

Contents of indole-3-acetic acid (IAA), gibberellin A_1_ (GA_1_), gibberellin A_4_ (GA_4_), *N*^6^-isopentenyladenine (iP), *trans*-zeatin (tZ), dihydrozeatin (DHZ), abscisic acid (ABA), salicylic acid (SA), jasmonic acid (JA), and jasmonoyl-l-isoleucine (JA-Ile) in flower buds of tomato were quantified by liquid chromatography–mass spectrometry (LC-MS). Swollen flower buds, which were expected to be blooming on the next day, were detached and weighed. Approximately 0.1 g (5–7 buds) of buds were merged in a group and immediately frozen in liquid nitrogen. Flower buds were ground with a mortar in liquid nitrogen. Hormones were extracted in 4 mL of extraction solvent [80% (v/v) acetonitrile, 19% (v/v) water, and 1% (v/v) acetic acid] supplemented with isotope-labeled internal standards as documented,^8^ at 4 °C for 1 h. Solid-phase extraction and LC-MS were performed as previously described.^9^

## 3 Results

### 3.1 Effect of Trehalose Foliar Application on Fruit Yield and Style Length of Eggplants

Our previous results in bell pepper (*C. annuum* L.) demonstrated that foliar application of trehalose increased fruit yield in the late autumn culture when bell pepper plants were threatened by low night temperature stress.^6^ In tomato, sudden temperature rise and soil dryness after or around the end of summer monsoon rains can cause blossom end rot and yield reduction.^10^ It often occurs in eggplant culture, too. Here, we examined the effect of foliar application of trehalose on fruit yield of eggplants grown in summer (Supplementary Figure S2).

Averages of fruit weight at the harvest were found to be larger in June and subsequently decreased over time (Supplementary Figure S2a). No apparent difference was seen between trehalose-treated and nontreated plants. Number of fruits per plant were relatively lower in June and increased in July and August in three eggplant cultivars (Supplementary Figure S2b), that showed a loose inverse correlation to fruit weight (Supplementary Figure S2a). It seemed that there is a slight increasing trend in fruit numbers in eggplants with trehalose treatment in August (Supplementary Figure S2b). However, this trend was not significant by analysis of variance (ANOVA) (*P* > 0.05). This may be attributed to the variation of environmental condition in the field and a modest heat stress on eggplants leading to yield loss with a nonsignificant level in this season.

In parallel, we examined occurrence of short-styled flowers in eggplants (Supplementary Figure S3). Weekly application of 0.05% and 0.1% of trehalose significantly increased the occurrence of long-styled flower in cultivars ‘Kurowashi’ and ‘Ryoma’ in July in 2017 (Supplementary Figure S3a), when the summer monsoon rains were about to end. This was not consistent in the cultivar ‘Nagaokanaga’.

A significant increase in long-styled flower rate was seen at 0.1% trehalose in June, while the opposite trend was seen in July. In 2018, longer styled flowers were observed in cultivars ‘Senryo-nigou’ and ‘Ryoma’ treated with trehalose in July. On the other hand, the cultivar ‘Kurobee’ did not show an increase in long-styled flowers in July (Supplementary Figure S3b). Increase in long-styled flowers was not apparent in 2019 (Supplementary Figure S3c). Here, we observed that foliar application of 0.05% or 0.1% trehalose occasionally caused mitigation of short-styled flowers and promotion of long-styled flowers in eggplants, while it was not consistent through the study and may depend on cultivars and weather variation among seasons.

### 3.2 Foliar Application of Trehalose Promotes Style Elongation but Not Pollen Germination in Tomato

As the effects of trehalose foliar application gave variable results for fruit yield and style length in the field experiments of eggplants, we examined short-styled flower occurrence in model tomato plants, cultivar ‘Micro-Tom’, which were cultured in an environment-controlled growth chamber. ‘Micro-Tom’ is known for its suitability to be cultured in a plant growth chamber until seed-setting stage due to its small size and relatively low light intensity requirement.^11^ Typically, tomato flowers started to bloom at the 5th week after sowing in the experimental conditions in this study. We examined the lengths of stamen and style and the height of ovary (Figure 1a) at the 5th to 8th weeks after seed sowing. In the control condition, ovary height showed an increasing trend from 1.29 ± 0.09, 1.37 ± 0.07, and 1.52 ± 0.06 mm (mean ± standard error) at the 5th week to 1.63 ± 0.03, 1.70 ± 0.03, and 1.71 ± 0.04 mm at the 8th week along with fruit development (Figure 1b). Stamen length became shorter once at the 6th week (6.12 ± 0.06, 6.03 ± 0.07 and 6.14 ± 0.08 mm) and returned to the level of the 5th week by the 8th week (6.27 ± 0.05, 6.34 ± 0.03, and 6.40 ± 0.05 mm) (Figure 1c). Changes in ovary height and stamen length over time were not affected by trehalose treatment. Style length decreased at the 6th week and was kept at a similar length at least until the 8th week in the control condition (4.83 ± 0.28 mm, mean ± standard error) (Figure 1d). These changes in floral organ morphology consequently rendered the pistil/stamen ratio slightly lower over time in the control condition (Figure 1e). On the other hand, style lengths of trehalose-treated plants showed an increasing trend at the 7th week for 0.1% (w/v) trehalose treatment (4.94 ± 0.08 mm) and at the 7th and 8th weeks for 0.5% (w/v) trehalose treatment (5.02 ± 0.10 and 5.12 ± 0.08 mm, respectively) (Figure 1d). As a result, the pistil/stamen ratio in trehalose-treated plants was significantly greater at the 7th week for 0.1% trehalose and at the 7th and 8th weeks for 0.5% trehalose (Figure 1e), suggesting that foliar application of trehalose promoted style length in tomato seedlings. The numbers of short-, medium-, and long-styled flowers were grossly counted throughout the experiments. Agreeing with the observation in pistil/stamen ratio, the rate of long-styled flowers increased by foliar application of 0.5% trehalose to tomato plants (Table 1).

**Table 1:** Number of Short-, Medium-, and Long-Styled Flowers in Tomato Cultivar ‘Micro-Tom’

	number of observed styles^a^			
trehalose (%)	long	medium	short	χ^2^ test (*P*)
0.0	2 (2.2)	81 (91.0)	6 (6.7)	0.10
0.1	5 (6.3)	69 (86.3)	6 (7.5)	0.71
0.5	13 (21.3)	45 (73.8)	3 (4.9)	<0.01

a Occurrence in percentage is designated in parentheses.

Pollen germination rate is an important feature for successful pollination. Here, we questioned whether trehalose affects pollen germination rate. The germination rates of tomato pollen of which seedlings were foliar sprayed with 0.0, 0.1, and 0.5% (w/v) trehalose were found to be of similar levels at around 80–90% (Supplementary Figure S4). Taken together with the previous report, we infer that the improvement of crop yield by foliar trehalose application reported in bell pepper^6^ is, at least in part, attributable to the prevention of short-styled flower rather than enhancement of pollen germination rates.

### 3.3 Effect of Other Sugar Species on Style Elongation

**Figure 2:**
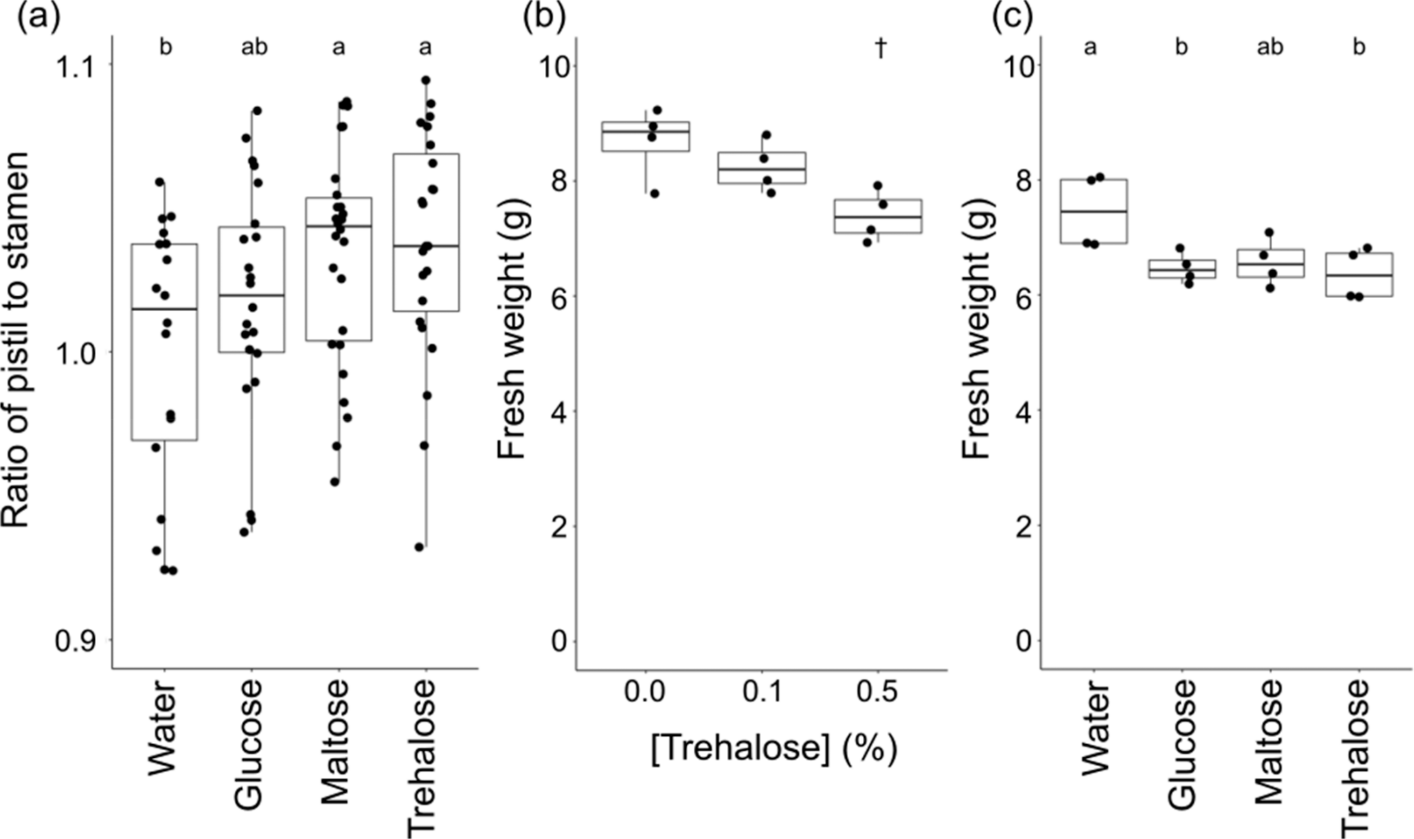
Effects of foliar application of sugar species on the pistil/stamen ratio and fresh weight of tomato shoot (cultivar ‘Micro-Tom’). Data are shown by box plots. (a) Effects of foliar application of glucose, maltose, and trehalose on the pistil/stamen ratio. Small letters indicate significant difference between treatment (Tukey–Kramer, *P* < 0.05). Concentration of sugars was 0.5% (w/v). Effect of (b) trehalose and (c) other sugars (0.5% w/v) on the fresh weight of shoots of tomato (*n* = 14–22 independent flowers). Data was collected on the 7th week. The dagger indicates significant difference from the control (0.0%) (Dunnett’s test, *P* < 0.05, *n* = 4). Small letters indicate significant difference between treatment (Tukey–Kramer, *P* < 0.05).

To gain insight into sugar species specificity for the style elongation action, in addition to trehalose, we examined the effect of glucose and maltose on the ratio of pistil to stamen length at the 7th week (Figure 2a). Treatment with 0.5% (w/v) glucose did not promote the pistil/stamen ratio as compared to the water control. Maltose [0.5% (w/v)] promoted the ratio to comparable levels as trehalose. Taken together, treatment with disaccharide consisting of two glucose molecules, i.e., trehalose and maltose, is able to promote style elongation, even though the chemical bonds between the glucose molecules in both disaccharides are distinct.

### 3.4 Effect of Trehalose and Other Sugars on the Growth of Shoots

During the experiments above, we noticed that foliar application of trehalose showed a detrimental effect on the plant size. To investigate further, we examined the effect of trehalose on fresh weight of shoots at the 8th week. Fresh weight of shoots treated with 0.5% trehalose was reduced by 15% (Figure 2b). Interestingly, other sugars, glucose and maltose, showed a similar detrimental effect on shoot growth (Figure 2c). This indicates that the adverse effect of sugars on shoot growth is mechanistically distinct from the action of disaccharides in promoting pistil lengths.

### 3.5 Trehalose Exhibits a Style Elongation Effect through Roots/Soil

**Figure 3:**
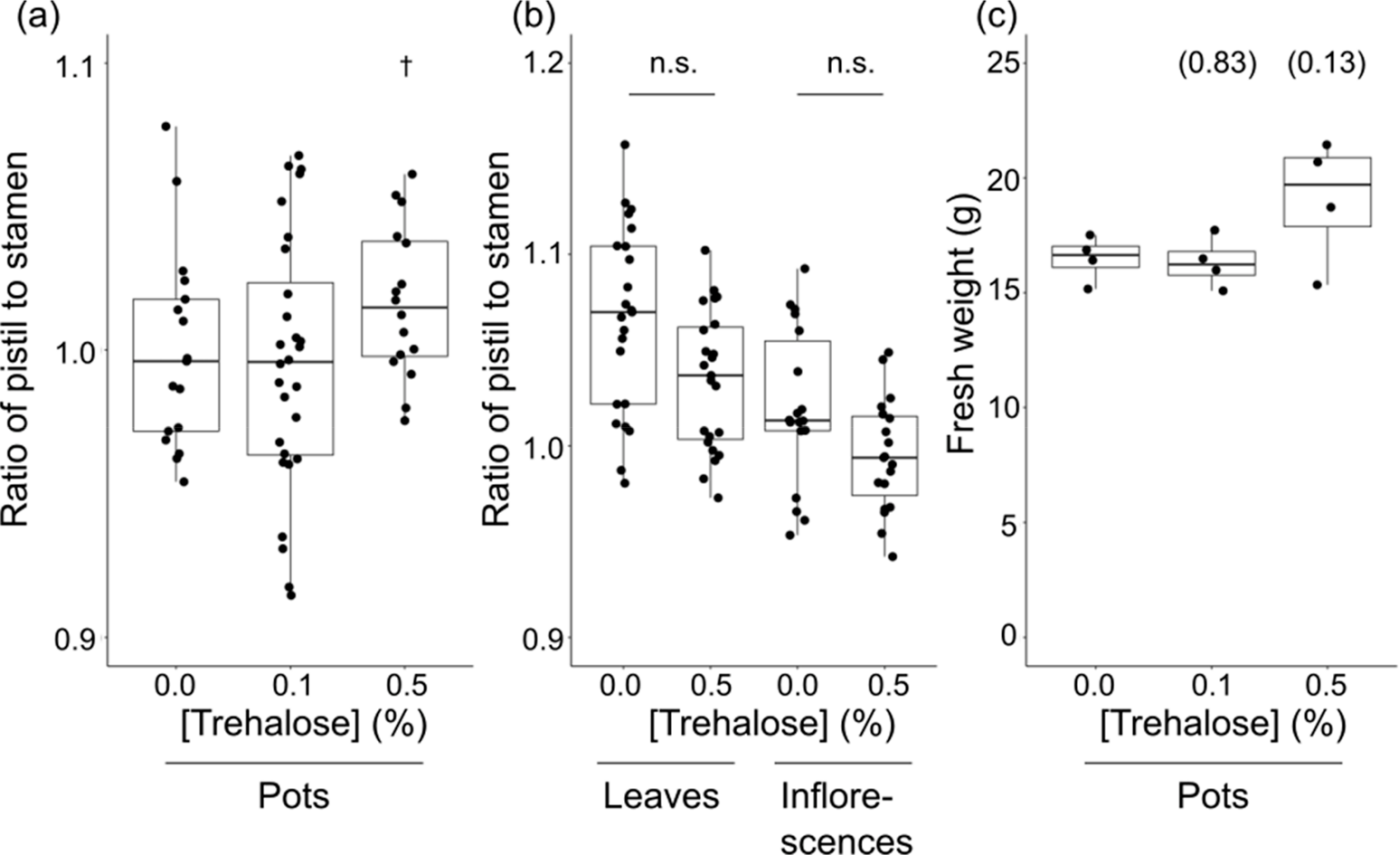
Effect of trehalose application to soil, leaves, and inflorescences on the ratio of pistil length to stamen length of tomato cultivar ‘Micro-Tom’. Data are shown by box plots. (a) Effect of trehalose application to soil on the pistil/stamen ratio. Data were collected on the 7th week. The dagger indicates significant difference from the control (0.0%) (Dunnett’s test, *P* < 0.05). (b) Effect of trehalose application on leaves and inflorescence on the pistil/stamen ratio. Leaves or inflorescence were dampened with distilled water (0.0%) or trehalose solution (0.5%) using a paint brush so that the treated surface appeared similar to that of foliar application using a sprayer. The surface of the soil was covered with a plastic wrap during the treatment to avoid any dropping of the solution to contact with the soil. n.s. indicates not significant by Student’s *t*-test. (c) Effect of trehalose application to soil on the fresh weight of shoots. Data were collected on the 8th week. Numbers in parentheses indicate *P* value examined by Tukey-honestly significant difference test (*n* = 4).

In agronomical practices, both foliar application [at 0.05–0.1% (w/v)] and irrigation [at 0.06–0.1% (w/v)] are utilized for trehalose application according to user’s manuals of the agricultural materials provided by the manufacturers, TreAce (Takii Seed Co. Ltd., Kyoto), TrehaMonogatari (Kawai Hiryo, Shizuoka, Japan), and TrehaTop (DAN Chemical Co. Ltd., Fujieda, Japan). Here, we noticed that soil in the pot was unintentionally moistened with solution dropping from the shoots during foliar application. We questioned whether trehalose affects the style length through intake via the leaves and/or through the soil. Approximately 5 mL of trehalose solution was carefully applied directly on the soil avoiding a direct contact of trehalose to the shoots as described in Materials and Methods. The ratio of pistil length to stamen length was greater in the tomato plants of which 0.5% (w/v) trehalose solution was added to the soil (Figure 3a). Meanwhile, leaves or inflorescence was carefully dampened with 0.5% trehalose solution once a week using a paint brush. Careful precautions were taken to avoid dropping of the solution on the soil so that the surface of leaves and inflorescence appeared similarly wet as the sprayed shoots in foliar treatment. Treatments to the shoots did not result in longer pistils than the control, but rather shorter than the stamen (Figure 3b). In contrast to foliar application, irrigation did not show a detrimental effect on the fresh weight (Figure 3c). These results suggest that trehalose promotes style elongation through the root system or the soil environment but not through the absorption by the surface of the shoots.

### 3.6 Hormone Contents in the Flower Buds of Tomato

The roles of plant hormones on style growth have long been acknowledged.^12,13^ Here, we examined the contents of ten plant hormones in swollen flower buds by LC-MS to seek potential mediators for style elongating action of trehalose (Supplementary Figure S5). In the flower buds of tomato, GA_4_ and DHZ were below the detection limits (∼10 pg per sample). Contents of GA1, IAA, tZ, iP, ABA, JA, and JA-Ile did not show any significant difference among treatments (Supplementary Figure S5a–g). SA contents decreased in flower buds collected from tomato seedlings sprayed with 0.1% (w/v) trehalose. However, 0.5% (w/v) trehalose did not cause a decrease in SA (Supplementary Figure S5h), although trehalose application at both concentrations enhanced pistil/stamen ratio in tomato (Figure 1e). This suggests that the change in SA contents is not coinciding with the style elongation effect. Consequently, we did not find any clue suggesting the involvement of plant hormones in the style-elongation effect of trehalose in this study.

## 4 Discussion

At present, the physiological role of trehalose in plants is poorly understood. The reports for physiological effects of exogenous trehalose on plants are, so far, inconsistent. Trehalose was reported to enhance plant resistance against biotic and abiotic stresses, while it inhibits growth.^5^ It was also reported to control inflorescence architecture in maize.^14^ In agricultural practices, trehalose is often utilized aiming at promotion of stress resistance in crops. Although its benefit has been empirically acknowledged by farmers, the physiological bases of externally applied trehalose to crop production and/or stress resistance has remained to be elucidated. We examined the effect of trehalose on fruit yield of eggplants in summer culture. However, we did not observe significant effect of trehalose application on the yield (Supplementary Figure S2). Stresses in summer culture of eggplants in the year might not be so severe to impose significant effect on crop production.

On the other hand, we showed that foliar application of trehalose reduced the occurrence of short-styled flowers in eggplants (Supplementary Figure S3) and tomato (Table 1; Figure 1). To our best knowledge, this is the first study describing the action of trehalose mitigating short-styled flower of plants. This finding is consistent with our previous finding that trehalose application increased fruit yield of bell peppers, which were cultured under a low night temperature condition in a late autumn culture.^6^

Floral organ development is related to sugar supply from the source tissue.^15^ In plants, trehalose is synthesized by a two-step reaction comprised of (i) the production of trehalose-6-phosphate (T6P) from uridine-5′-diphosphate glucose and glucose-6-phosphate by T6P synthase and (ii) dephosphorylation of T6P to trehalose by T6P phosphatase.^16^ T6P acts as a specific signal for sucrose availability through inhibiting the activity of Sucrose non-Fermenting Related Kinase 1 (SnRK1).^17^ Exogenous application of trehalose causes an increase of the precursor, T6P, that would imitate rich sucrose availability.^17^ Perturbation of sucrose signaling by exogenous trehalose, which would cause an alteration of T6P levels, may cause elongation of style under a stress with insufficient sucrose availability. The molecular basis of the style elongating action of trehalose in Solanaceae remained to be further studied.

Maltose and trehalose are disaccharides consisting of two glucose molecules linked by an α-1,4-glycosidic bond and a 1,1-α,α-glycosidic bond, respectively. Both sugars showed style elongation action in tomato (Figure 2). The monosaccharide glucose did not show the same action as these disaccharides. Therefore, it is unlikely that maltose and trehalose exhibit the action through decomposition to glucose. It remains unknown if maltose and trehalose have a common mode of action or a distinct mechanism. It also remains unanswered whether oligosaccharides longer than two sugars and/or hetero-disaccharides would show a similar effect.

Against our initial expectation, trehalose imposes a style elongation effect through irrigation via roots but not via direct application to shoots (Figure 3). The observed effect from foliar application of trehalose could be attributed to a considerable amount of dripped trehalose solution from the shoots to the soil. It remains elusive whether trehalose acts directly to the roots or indirectly mediated by the soil matrix or microbes in the rhizosphere. Given that trehalose is absorbed by the root directly, it is unraveled whether trehalose reaches to the inflorescence as it is or is converted to a systemic signal in the roots. Regardless of the mode of action of trehalose after its uptake through the root, our finding provides valuable information on an effective method of trehalose application to crops–through irrigation.

Trehalose has been reported to have an adverse effect on shoot growth in *Cuscuta reflexa* and *Arabidopsis thaliana*.^18−20^ In this study, it was shown that an adverse effect of vegetative growth was not specific to trehalose but was also observed with glucose and maltose (Figure 2). Here, we observed that a topical application of trehalose to shoots caused only growth inhibition but not style length promotion. This suggests that trehalose should be applied by irrigation to Solanaceae crops if it was aiming at improvement of pollination rate.

Short-styled flowers are an agricultural problem since they can result in low fertility and yield reduction in solanaceous crops. In the case of tomato and eggplants, farmers often conduct parthenocarpy using agrochemicals to set fruits without pollination. On the other hand, a successful pollination is a prerequisite for production of a normal (commercially valuable) shaped fruit in bell pepper. Trehalose application is found to be capable of preventing yield loss caused by stress-induced short-style flower in bell peppers, eggplants, and tomato cultivations without parthenocarpy practices. This demonstrates its potential as a biostimulant.

It was shown that maltose and trehalose have a similar effect on style elongation (Figure 2). Since maltose is generally less stable in the field as compared to trehalose, biochemical stability of trehalose would be advantageous in a field situation. Nevertheless, we observed variations in the effect of trehalose among cultivars and environments in field experiments of eggplants. Conditions and periods of trehalose application should be further examined for practical application.

## Supporting Information Available

Classification of eggplant flowers according to style length morphology (Figure S1); effects of foliar application of trehalose on average fruit weight and fruit number in eggplants (Figure S2); effect of foliar application of trehalose on style length in eggplants (Figure S3); pollen germination rate in ‘Micro-Tom’ tomato sprayed with trehalose (Figure S4); effect of foliar application of trehalose on plant hormone contents in swollen flower buds of ‘Micro-Tom’ tomato (Figure S5) (PDF)
