# Supplementary material for: Application of Trehalose Mitigates Short-Styled Flowers in Solanaceous Crops
Source: J Agric Food Chem. 2023 Apr 3;71(14):5476–82. doi: 10.1021/acs.jafc.2c08479 (PMC10103160; doi:10.1021/acs.jafc.2c08479)
Supplement: Supplementary file 1 — jf2c08479_si_001.pdf [file jf2c08479_si_001.pdf]

Supporting Information

Application of trehalose mitigates short-styled flowers in Solanaceous crops

Izumi C. Mori[1,*], Takakazu Matsuura[1], Masahiro Otao[2], Lia Ooi[2], Yasuyo Nishimura[3], and Takashi Hirayama[1]

[1] Institute of Plant Science and Resources, Okayama University, Kurashiki, 710-0046, Japan;

[2] Research Department, Hayashibara Co., Ltd., 675-1 Fujisaki, Naka-ku, Okayama 702-8006, Japan;

[3] Faculty of Agriculture and Marine Science, Kochi University, Nankoku, 783-8502, Japan

* Corresponding author: I. C. Mori, imori@okayama-u.ac.jp

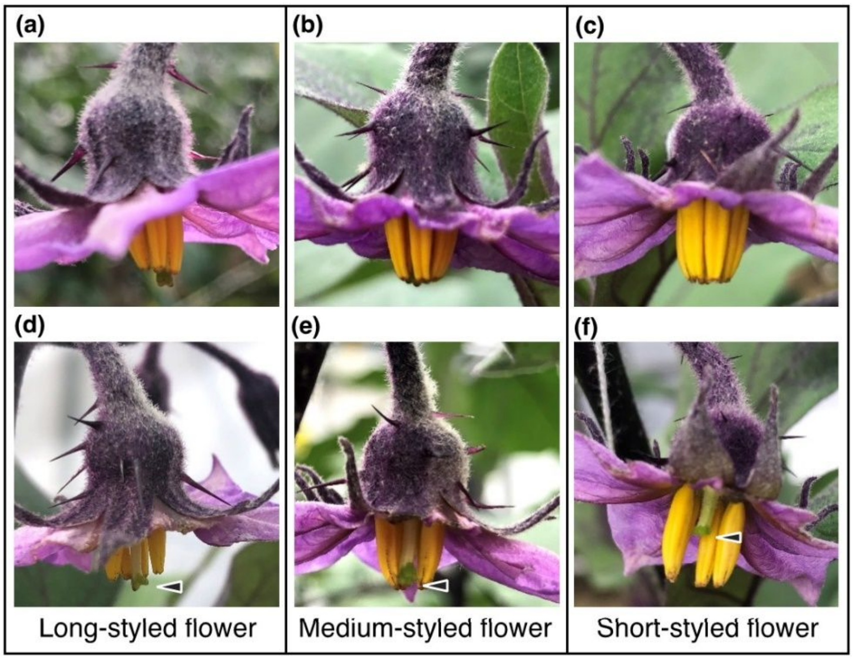

Supplementary Fig. S1. Classification of eggplant flowers according to style length morphology. (a) and (d), Long-styled flower. (b) and (e), Medium-styled flower. (c) and (f), Short-styled flower. (d), (e) and (f), flowers of which anthers are partly removed to show the pistil in the anther cone. Arrowheads indicate the position of the tip of the pistil. Flowers were of cultivar 'PC Oryo' cultivated on Monobe campus, Kochi University.

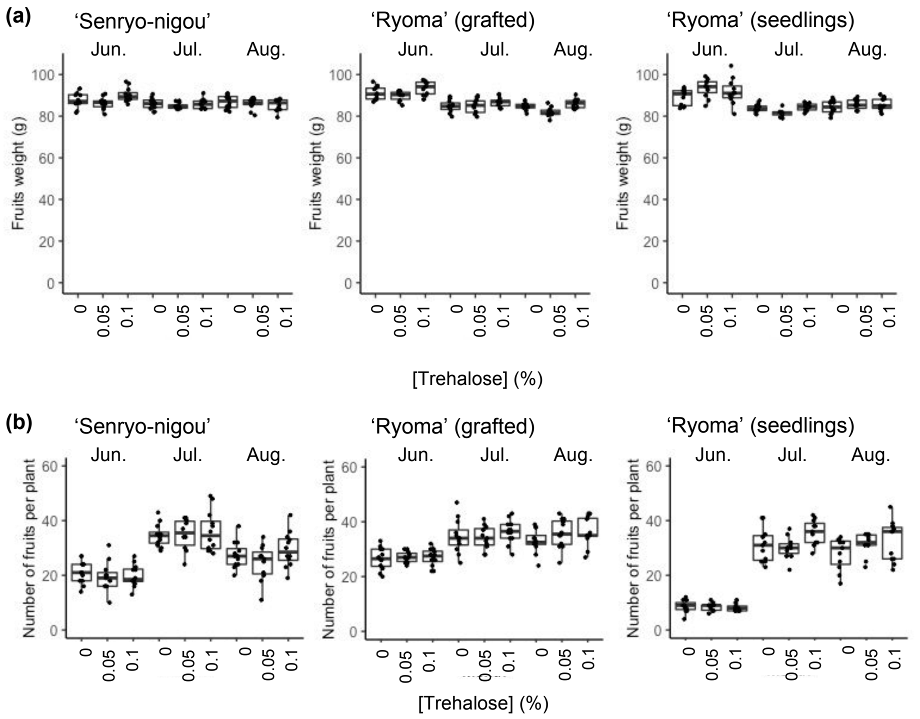

Supplementary Fig. S2. Effects of foliar application of trehalose on average fruit weight and fruit number in eggplants. Data were acquired from 10 – 12 plants in 2019. There was no significance in all experiments (Dunnett's test, $\alpha = 0.05$).

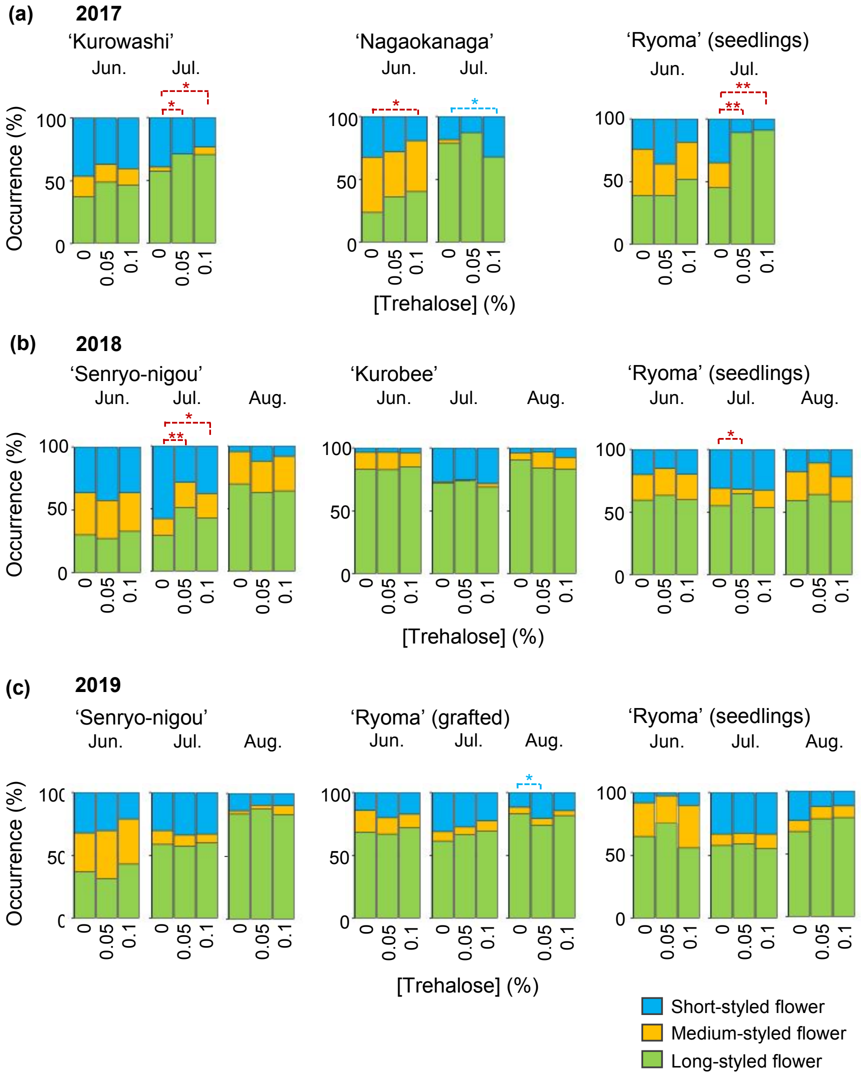

Supplementary Fig. S3. Effect of foliar application of trehalose on style length in eggplants. Length of styles were visually classified as short-styled, medium-styled, and long-styled as described in Materials and Methods. Ten to twelve plants were examined through the season. * and ** indicate significance at $P < 0.05$ and <0.01, respectively (χ square test). Red and blue symbols indicate significance with increased and decreased values, respectively.

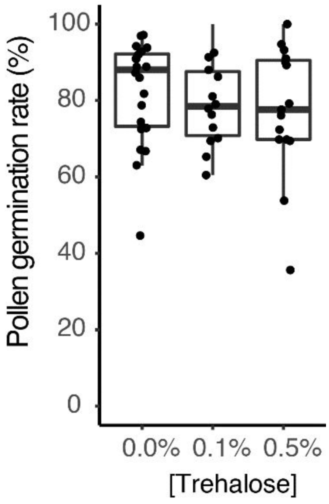

Supplementary Fig. S4. Pollen germination rate in 'Micro-Tom' tomato sprayed with trehalose. The data collected in the 7th week is shown by box plots. There was no significant difference between treatments (Tukey-Kramer, $P < 0.05$).

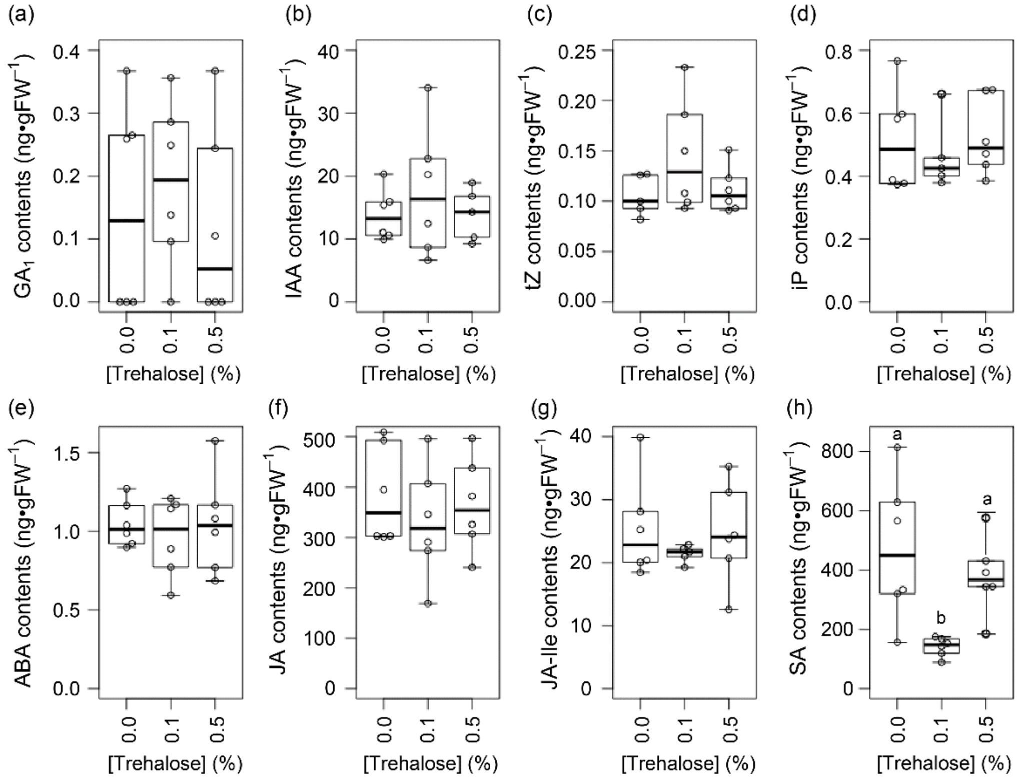

Supplementary Fig. S5. Effect of foliar application of trehalose on plant hormone contents in swollen flower buds of 'Micro-Tom' tomato. Data were shown by box plots (n = 6). Small letters indicate significant difference between treatment (Tukey-Kramer, $P < 0.05$).
